# Altemeier’s procedure for complete rectal prolapse; outcome and function in 43 consecutive female patients

**DOI:** 10.1186/s12893-018-0463-7

**Published:** 2019-01-03

**Authors:** Mario Trompetto, Roberta Tutino, Alberto Realis Luc, Eugenio Novelli, Gaetano Gallo, Giuseppe Clerico

**Affiliations:** 1 Department of Colorectal Surgery. S Rita Clinic, Vercelli, Italy; 2 Dept. of Surgical, Oncological and Stomatological Disciplines, University of Palermo, Palermo, Italy; 3 Department of Biostatistics, S.Gaudenzio Clinic, Policlinico di Monza, Italy; 4 Department of General Surgery, University of Catanzaro, Catanzaro, Italy

**Keywords:** Rectal prolapse, Altemeier procedure, Perineal rectal resection, Pelvic floor disorders, Pelvic organ prolapse, Fecal incontinence, Urinary incontinence

## Abstract

**Background:**

The aim of this retrospective study was to evaluate morbidity, mortality, postoperative function and recurrences in patients treated by Altemeier’s rectosigmoidectomy for complete rectal prolapse in a referral center for pelvic floor functional disorders.

**Methods:**

Peri-operative data on 43 consecutive female patients were reviewed. At follow-up any change in pelvic floor function and recurrences were determined. Thirty four patients were assessed at a median interval of 49 (2–135) months, six being deceased for reason not related to the prolapse and three lost to follow-up.

**Results:**

Post-operative complications at 30 days occurred in 18 patients (38%). Major complication occurred in only one patient that was pneumonia with lung failure. Major complications were not related to the ASA score, BMI or age [average age 76.4]. There was no post-operative mortality at 30 days.

At long-term follow-up functional results demonstrate a statistically significant decrease in the Obstructive Defecation Syndrome (ODS) score, but no statistically significant changes in the Vaizey score, the International Consultation on Incontinence Questionnaire Short Form (ICIQ-SF) score and the urinary retention score. ODS score decreased with respect to levatorplasty and the change was statistically significant instead of Vaizey score in which were not.

At the same follow-up there were 12 (35%) cases of recurrence with an estimated risk at 48 months of 40%. There were no statistically significant differences between patients with and without recurrence regarding age (*p* = 0.188), BMI (*p* = 0.864), ASA score (*p* = 0.433), previously repaired prolapse (*p* = 0.398), previous hysterectomy (*p* = 0.705), length of resected bowel (*p* = 0.126), and levatorplasty (*p* = 0.304). Patient satisfaction showed a mean of 8.8 and 6.4 respectively in patients without and with recurrences (*p* = 0.012).

**Conclusions:**

Altemeier’s procedure had in our series low complications rate and no mortality. It offered improved evacuation in constipated patients while didn’t improve fecal and urinary continence. Recurrence of prolapse was 40% at four years.

**Electronic supplementary material:**

The online version of this article (10.1186/s12893-018-0463-7) contains supplementary material, which is available to authorized users.

## Background

Rectal prolapse has an estimated incidence of 2.5/100000 of the general population. It occurs mostly in patients over 50 years of age with a female/male ratio of around 10/1 [1]. The etiology is multifactorial and includes weakness of the pelvic floor, chronic constipation, multiple pregnancies, previous pelvic surgery and a deep pouch of Douglas [2]. It is also associated with a mixed pattern of functional disorders ranging from difficulty of evacuation of stool, so called obstructive defecation syndrome (ODS), to fecal incontinence.

The aim of surgical repair is to reduce the mobility of the rectum and sigmoid colon by fixation with or without removal of the prolapsing rectum and sigmoid colon and to give mechanical support to sphincters and pelvic floor [3].

Despite anatomical correction by surgery, patients frequently complain persisting pelvic floor symptoms and recurrences.

Surgical treatments proposed are divided in abdominal and perineal procedures.

Altemeier’s procedure is one of the well-known perineal operations to treat full-thickness rectal prolapse; it removes the prolapse without a pexy and performs only a partial reconstruction of the pouch of Douglas.

In the present study we evaluated the results of Altemeier’s procedure in a sequential series of patients with complete rectal prolapse to determine the rates of early morbidity and mortality, the long term functions and recurrences.

## Methods

Data on 43 consecutive female patients undergoing Altemeier’s procedure for complete rectal prolapse were reviewed. The patients were identified by the diagnostic code on admission of International Classification of Diseases (ICD)-9: 569.1 and by the surgical code ICD-9: 4849.

Demographic data including age, number and type of delivery, comorbidity, previous pelvic or perineal surgery, duration of symptoms, bowel function including frequency of defecation, urgency and incontinence, urinary function, body mass index (BMI) and American Society of Anesthesiologists (ASA) score were recorded.

Data on perioperative management including bowel preparation, antibiotic and thromboembolic prophylaxis, and type of anesthesia were also collected.

The surgical technique including the addition of levatorplasty to the rectosigmoidectomy, duration of the operation, the length of resected bowel, the interval from operation to the first bowel movement and the length of hospital stay were all recorded.

30 days morbidity according to Clavien-Dindo classification [4] and 30 days mortality were recorded.

Long term follow-up was performed in 34 available patients with three patients lost to follow up and six deceased for reasons related to their ages and comorbidity not related to the surgical procedure (they would have had at the time of long term follow-up an average age of 90 years old with a median of 91 years old) being excluded from the analysis. Unfortunately, we have no data on their recurrence state.

Functional results analyzing bowel and urinary function patient satisfaction were investigated.

All patients were classified using the ODS score described by Altomare et al. [5]. Continence was assessed pre and post-operatively using the Vaizey scoring system [6], which ranges from 0 (normal continence) to 24 (severe incontinence). Urinary function was determined pre and post-operatively using the validated International Consultation on Incontinence Questionnaire Short Form (ICIQ SF) score (range 0 [normal]-21) and a pre and post-operative evaluation of the residual urinary volume was made by a four-degree severity score (0 for < 50 mL, 1 for > 50 < 100 mL, 2 for > 100 < 200 mL, 3 for > 200 ml) [7, 8].

Patient satisfaction was determined using a simple numerical scale from 0 (not satisfied) to 10 (completely satisfied).

Endoanal ultrasound (EUS), contrast defecography, magnetic resonance imaging (MRI)- defecography, colonic motility and anorectal manometric studies were not routinely performed in all patients, usually owing to their advanced age and the obvious diagnosis of rectal prolapse on observation. Recurrence of the prolapse was analyzed.

Statistical analysis: Descriptive data are presented as parametric data and non-parametric data. The relationship between post-operative complications and age, ASA and BMI was analyzed using the unpaired t-test.

Comparison between pre-operative and post-operative functional scores was performed using the paired t-test or Wilcoxon’s rank sum test for paired data.

The relationship between changes in the ODS score and Vaizey score in respect to levatorplasty was evaluated using the unpaired t-test and the Mann-Whitney U-test.

The probability of recurrence at 48 months was determined using the Kaplan-Meier method. The relationship between recurrence and age, BMI, previous rectal prolapse surgery, previous hysterectomy, levatorplasty, length of resected bowel and gender was evaluated using an independent-sample t-test, Pearson’s chi-squared test or Fisher’s exact test. The Mann-Whitney U-Test was used to evaluate patient satisfaction regarding recurrence. A *p*-value of < 0.05 was considered to be statistically significant.

Statistical analysis was conducted using SPSS software (SPSS, Chicago, Illinois, USA) and MedCalc Statistical Software (MedCalc Software, Ostend, Belgium).

## Results

Forty-three female patients (mean age 76.4 ± 10 years) underwent Altemeier’s procedure between 2004 and 2015. Each female had had a mean of 1.4 deliveries. Twenty-eight (65%) patients had a previous history of cardiovascular disease, 13 (30%) a neurological or psychiatric disorder, and 30 (70%) had had previous pelvic surgery. Of these 30, 14 had had a previous surgical repair for rectal prolapse by various techniques (4 Delorme, 2 STARR, 1 transanal proctopexy, 1 rectosigmoidectomy + anal encirclement, 1 rectopexy, 1 rectopexy with mesh, 1 Wells’ procedure, 3 no data), 24 had had a hysterectomy and seven had had a cystopexy. The average duration of symptoms was 2 years. The average BMI was 22,2 (± 4.4). The ASA score was I [6 patients], II [21], III [15] and IV [1]. The mean preoperative scores for constipation and incontinence, the ICIQ SF score and preoperative residual urinary volume score are given in Table 1.

**Table 1:** Change in functional scores before and after Altemeier’s procedure

Global pelvic health	Pre-op (Mean ± SD)	Post-op (Mean ± SD)	*p*-value
ODS score	7.4/5.8	4.2/4.9	0.0004
Vaizey score	8.8/7.2	8.8/7.2	1.0000
ICIQ SF score	4.4/5.7	5.0/6.1	0.0625
Urinary retention	0.1/0.6	0.1/0.2	1.0000

### Surgery

All patients received a complete bowel preparation, antibiotic prophylaxis (Cefazolin and Metronidazole) and thromboembolic prophylaxis (low-molecular-weight heparin). General anesthesia was used in 18 (42%) patients and a spinal block in 25 (58%). The median duration of the surgical procedure was 69 (50–125) minutes. All patients had a coloanal hand sewn anastomosis and in 25 (58%) a levatorplasty was also performed. The median length of the resected bowel was 20 (12–70) centimeters.

Postoperatively the first defecation occurred at 24/48 h in 27 (63%) patients, at 72 h in 10 (23%) and on the fourth-sixth post-op day in 6 (14%). The mean length of hospital stay was 6 [3–8] days.

Post-operative complications at 30 days occurred in 18 patients (38%): these were classified as Clavien-Dindo grade 1 in 14 patients (78%), grade 2 in 3 patients (17%), grade 3 in zero, and grade 4 in only one patient (5%). Grade 1 and 2 were a minimal anastomotic leakage successfully treated conservatively, four post-operative anemia requiring blood transfusion in two, eight fever, two transitory electrolyte disturbances and one urinary retention. Grade 4 occurred in 44-years old patient with an history of dementia, Parkinson, chronic bronchitis and recurrent ab aspiration pneumonias who presented with an aspiration pneumonia and lung failure. There was no post-operative mortality at 30 days.

### Follow-up

As previously reported, six patients were deceased and three patients were lost to follow up leaving 34 with a median follow-up of 49 (2–135) months.

The mean pre and post-operative scores for the various functional indices are shown in Table 1. (Additional file 1).

There was no difference in the Vaizey, ICIQ SF and urinary retention score.

**Fig. 1:**
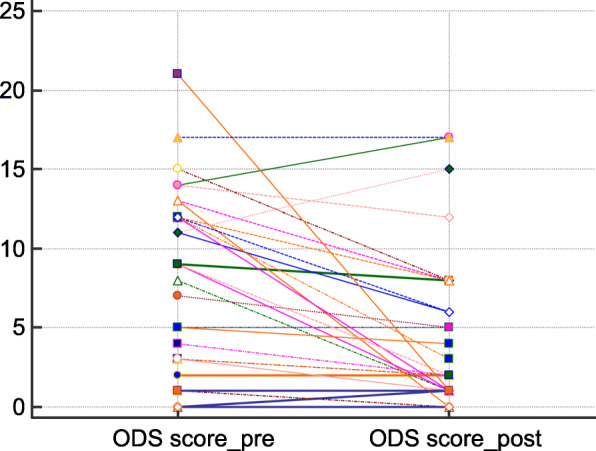
Comparison of the preoperative and postoperative obstructed defecation syndrome (ODS) scores. (Related-Samples Sign Test for paired data)

**Fig. 2:**
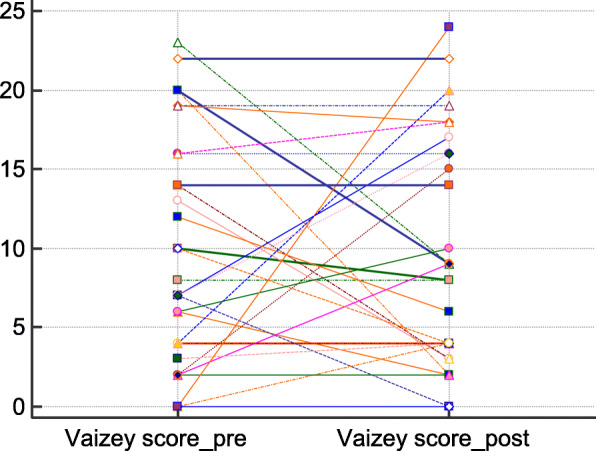
Comparison of the preoperative and postoperative Vaizey scores. (Wilcoxon signed rank test)

There was an improvement in the ODS score postoperatively in 21 of the 34 patients. Three patients experienced a worsening and in ten there was no change. The overall median decrease in ODS score was 1.5. There was a statistically significant decrease postoperatively in the median of the differences of 2.5 (*p* < 0.001) (Fig. 1). Fecal incontinence improved in 11 patients, worsened in 10 and was unchanged in 13. There was no statistically significant difference in the Vaizey score before and after surgery (*p* = 1.000) (Fig. 2).

The ICIQ SF score showed that urinary incontinence improved in one patient, worsened in five, and in 28 there was no change with a median pre-operative ICIQ SF score of 0 and no difference postoperatively (*p* = 0.062). One patient showed an improvement in urinary retention but in all other patients the score was unchanged (p = 1.000).

There was statistically significant differences in the ODS score changes between the 21 patients who underwent a levatorplasty and the 13 who did not with a median of differences of 0 in the group without plasty and of − 2 in the group with plasty (*p* = 0.0156) while there were no differences in Vaizey score changes (*p* = 0.4524).

**Fig. 3:**
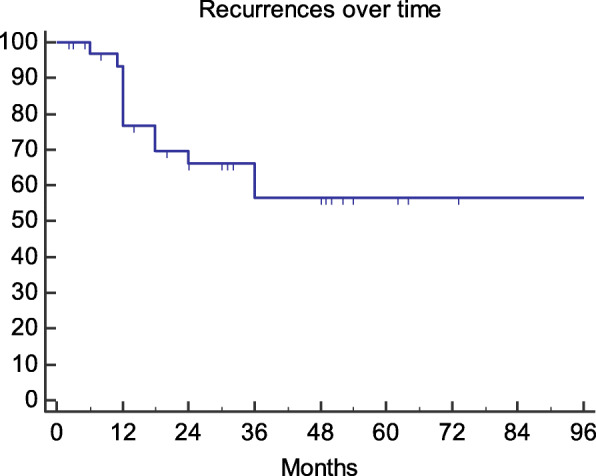
Recurrence over time (Kaplan-Meier curve)

During this period there were twelve cases (35%) of recurrence which resulted in a risk of recurrence at 48 months of approximately 40% (Fig. 3). The average time to recurrence was 17 months (SD 9.8- range 5–36). (Additional file 1).

There were no statistically significant differences between patients with and without recurrence regarding age (*p* = 0.188), BMI (*p* = 0.864), ASA score (*p* = 0.433), recurrent prolapse (*p* = 0.398), previous hysterectomy (*p* = 0.705), length of resected bowel (*p* = 0.126), and levatorplasty (*p* = 0.304) (Table 2).

**Table 2:** Possible factors related to recurrence

	Not Recurrence	Recurrence	*p*-value
Age Years (median, CI)	77.5 (72 to 85)	74.5(68 to 81)	0.188
BMI Kg/m2 (median, CI)	20.6 (19.9 to 22,0)	21.2(18.2 to 25.4)	0.864
ASA score (median)	2	2	0.433
Recurrent prolapse (No/Yes)	16/6	7/5	0.398
Previous hysterectomy (No/Yes)	8/14	3/9	0.705
Length of resected bowel cm (mean ± SD)	20.5/8.0	26.5/14.2	0.126
Levatorplasty (No/Yes)	7/15	6/6	0.304

Patient satisfaction showed a mean of 8.8 and 6.4 respectively in patients without and with recurrences (*p* = 0.012). Only two patients who presented with rectal prolapse recurrence underwent a reoperation, one redo-Altemeier’s procedure and one Goldberg’s procedure.

## Discussion

The present study evaluated the morbidity, mortality, function and recurrence rate in patients undergoing Altemeier’s operation for complete rectal prolapse.

Fleming et al. evaluated the perioperative outcome of patients with complete rectal prolapse from the American College of Surgeon National Surgical Quality Improvement Program (NSQIP) to determine the safety of different surgical approaches. They divided complications into minor and major, taking major complications to include organ space infection, cardiac and thromboembolic events, ventilator dependence, pneumonia, return to the operating room, renal failure and sepsis. Surgical site and urinary tract infection were considered to be minor. They found that a perineal approach was independently associated with a lower 30-day major and minor complication rate than any abdominal procedure. Resection-rectopexy had doubled the rate of complications than rectopexy alone [9]. These findings support the results obtained in the present study which included a rate of major complications of 2.3% (one patient), which were not related to the ASA score, BMI or age, and no 30 days mortality.

Altemeier’s procedure can be carried out under spinal anesthesia, avoiding the trauma of a laparotomy and permitting rapid recovery of alimentary function and mobility. Thereby it offers the advantages of minimal surgical stress and low post-operative morbidity and mortality. In literature morbidity ranges from 3 to 35% and mortality is very unfrequently reported (Table 3) [10–18]. Abdominal repair require general anesthesia and may contribute to the possible formation of pelvic adhesions, posing a potential risk of infertility in young female and of impotence in males with the addition of the risk of anastomotic leakage if a resection rectopexy is performed even if resection is nowadays seldom performed [19].

**Table 3:** Morbidity and mortality and functional results of Altemeier’s procedure in literature

Study	N. of patients	Morbidity	Mortality	Fecal continence	ODS
Kimmins (2001) [10]	63	10%	0	ND	ND
Cirocco (2010) [11]	103	14%	0	Improved	Improved
Lee (2010) [12]	143	13.8%	0	ND	ND
Ris (2011) [13]	60	35%	1.6%	62%	ND
Ding (2012) [14]	113	16.8%	ND	ND	ND
Senapati (2013) [15]	102	5%	2%	Improved	ND
Towliat (2013) [16]	26	ND	ND	ND	Worsened
Kim (2014) [17]	63	3%	1.6%	ND	ND
Elagili(2015) [18]	22	22%	0	Worsened	Worsened
Our series	43	2.3%	0	No change	Improved

A careful preoperative risk assessment of surgical and cardiopulmonary risks including ASA and functional status is mandatory to anticipate possible postoperative complications [20]. As previously suggested, patients with complete rectal prolapse should be preoperatively assessed holistically with a record made of fecal incontinence, constipation, dysuria or urinary retention and urinary incontinence [8].

The aim of surgical repair is to remove the prolapse, with the additional hope of restoring continence and relieve any evacuation difficulty with minimal morbidity and mortality [2, 21]. The attempt to improve function is based on the assumption that the restoration of the anatomy will lead to relief of disturbances of function [22].

Few publications reported data on the effect of Altemeier’s operation on function and those show different results among the series; data are summarized in Table 3 [10–18]. Many comparisons of the perineal and abdominal approaches have pointed to worsening or the de novo appearance of obstructed defecation in the case of the latter [19]. In contrast the perineal approach which reduces rectal capacity and rectal wall compliance may increase the frequency of defecation, urgency and fecal incontinence in up to 40% of patients [21] with constipation reported in 10% [22].

In our series although a statistically significant reduction in the ODS score was found, there was no change in any of the other parameters used to assess bowel and urinary function. Interestingly, levatorplasty offered an improvement in the ODS score while hadn’t any discernable effect on Vaizey score. Despite the finding of a higher satisfaction in all patients it is not surprising that this was largely due to the benefit perceived by the patients not developing recurrences.

Female gender with possible obstetric trauma, the wider pelvis and weaker pelvic floor due to age and gender are factors that would contribute to poor function and the failure of repair of the prolapse to alter most of the functional scores indicates that the prolapse itself may not be an important factor in the bowel and urinary dysfunction often observed in patients with prolapse.

Abdominal approaches have been shown to be associated with lower rates of recurrence than perineal procedures after which rates of up to 58% have been reported [19, 23]. Recurrences in our series occurred in 35% of cases, with an estimated risk of at 48 months of 40% (Table 4) [10–18, 24–30]. The high rate of recurrence at four years from surgery is likely to be multifactorial. Analysis of possible factors related to recurrence showed no statistical relationship to age, gender, BMI, ASA score, recurrent prolapse already repaired, previous hysterectomy, the length of resected bowel or the addition of a levatorplasty to the repair. This finding was in contrast to the findings of Ding et al. who reported a statistically significant association of revision Altemeier procedure with recurrence or to the report of Kim et al. who found that the removal of a shorter specimen was followed by a higher risk of relapse [14, 17]. In contrast our data were similar to those of Ris et al. who found no association between the length of the resected bowel and recurrence [13]. In Table 4 are summarized the literature data on recurrences after Altemeier’s procedure [10–18, 24–30].

**Table 4:** Recurrences after Altemeier’s procedure in literature

Study	n. patients	Recurrences (%)	Time to recurrence (years)
Altemeier (1972) [24]	106	2.8%	ND
Friedman (1983) [25]	27	50%	ND
Gopal (1984) [26]	18	5.5%	ND
Williams (1992) [27]	114	10%	1
Ramanujam (1994) [28]	72	5.5%	ND
Kimmins (2001) [10]	63	6.4%	2
Chun (2001) [29]	109	7.7% with levatorplasty	4
		20.6% without levatorplasty	1
Hammond (2007) [30]	48	16.7%	10
Cirocco (2010) [11]	103	0	ND
Lee (2010) [12]	143	11.4%	1
Ris (2011) [13]	60	14% actuarial	4
Ding (2012) [14]	113	18%	ND
Towliat (2013) [16]	26	26.9%	ND
Senapati (2013) [15]	102	41% actuarial	5
Kim (2014) [17]	63	13%	2
Elagili (2015) [18]	22	9%	1
Our series	43	40% actuarial	4

The relatively high number of recurrences after perineal repair should be balanced with the minimal invasiveness of the technique and the possibility of repeat it with no additional morbidity and considering the relatively long recurrence time. This may be further supported by the finding in the present study of an improvement in the ODS which will give some symptomatic relief.

In contrast to the many observational studies, the PROSPER randomized study, the largest on rectal prolapse, compared the recurrence rate, incontinence, bowel function and quality of life (QoL) of perineal and abdominal procedures and showed an improvement in symptom-specific and overall QoL for both types of procedure with a similar incidence of recurrence (28% vs 19%; *p* = 0.2) and no significant difference in bowel function and QoL [15]. Moreover, a recent Cochrane review failed to confirm the superiority of transabdominal over perineal procedures, due to the heterogeneity and poor quality of the available studies [31].

To note that in contrast to the reports of open abdominal corrections of the prolapse, laparoscopic ventral rectopexy is actually largely spread and it showed comparable morbidity and lower mortality rates, improved short term outcomes and shorter hospital stay than perineal surgery and moreover less morbidity in comparison to the open abdominal procedures [32–36]. Furthermore functional outcomes (constipation, continence and outlet obstruction) after laparoscopic ventral rectopexy were at least equivalent as the ones after open abdominal or perineal procedures [36, 37]. These interesting results are actually changing the attitude toward a use of this minimal invasive abdominal technique in the management of full thickness rectal prolapse for all patients.

The present study has a number of limitations. It was retrospective and the follow up was not performed in all patients. The score on patient’s satisfaction and the urinary retention score are not validated.

## Conclusions

The Altemeier’s procedure is an available low risk treatment that can be performed under regional anesthesia, recovery is rapid and it gives immediate relief of the prolapse itself. So, it could be an available option for frail patients with complete rectal prolapse. The relatively high number of recurrences should be balanced with the minimal invasiveness of the technique and the possibility of repeating it with no additional morbidity and considering the relatively long recurrence time that in our cases was 17 months in mean with no deterioration in function.

## Additional file

Additional file 1: Data on follow-up and recurrences. Pre and post-operative functional scores and data above recurrences and time to recurrences collected from each patient. (XLSX 20 kb)

## Abbreviations

ACS NSQIP: American College of Surgeon National Surgical Quality Improvement Program
ASA score: American society of anesthesiologists score
BMI: Body mass index
ICD-9: International Classification of Diseases- 9
ICIQ-SF Score: International Consultation on Incontinence Questionnaire Short Form score
ODS: Obstructed defecation syndrome
QoL: Quality of life
